# Probio-X Relieves Symptoms of Hyperlipidemia by Regulating Patients’ Gut Microbiome, Blood Lipid Metabolism, and Lifestyle Habits

**DOI:** 10.1128/spectrum.04440-22

**Published:** 2023-04-06

**Authors:** Huan Wang, Cuicui Ma, Yan Li, Lei Zhang, lima A, Chengcong Yang, Feiyan Zhao, Haifeng Han, Dongyang Shang, Fan Yang, Yuying Zhang, Heping Zhang, Zhihong Sun, Ruifang Guo

**Affiliations:** a Department of Clinical Nutrition, Inner Mongolia People’s Hospital, Hohhot, Inner Mongolia, China; b Inner Mongolia Key Laboratory of Nutrition and Health, Inner Mongolia People’s Hospital, Hohhot, Inner Mongolia, China; c Inner Mongolia Key Laboratory of Dairy Biotechnology and Engineering, Inner Mongolia Agricultural University, Hohhot, Inner Mongolia, China; d Key Laboratory of Dairy Products Processing, Ministry of Agriculture and Rural Affairs, Inner Mongolia Agricultural University, Hohhot, Inner Mongolia, China; e Key Laboratory of Dairy Biotechnology and Engineering, Ministry of Education, Inner Mongolia Agricultural University, Hohhot, Inner Mongolia, China; Tainan Hospital, Ministry of Health and Welfare

**Keywords:** hyperlipidemia, probiotics, gut microbiota, carnitine, lipid metabolism

## Abstract

Hyperlipidemia is a key risk factor for cardiovascular disease, and it is associated with lipid metabolic disorders and gut microbiota dysbiosis. Here, we aimed to investigate the beneficial effects of 3-month intake of a mixed probiotic formulation in hyperlipidemic patients (*n* = 27 and 29 in placebo and probiotic groups, respectively). The blood lipid indexes, lipid metabolome, and fecal microbiome before and after the intervention were monitored. Our results showed that probiotic intervention could significantly decrease the serum levels of total cholesterol, triglyceride, and low-density lipoprotein cholesterol (*P < *0.05), while increasing the levels of high-density lipoprotein cholesterol (*P < *0.05) in patients with hyperlipidemia. Probiotic recipients showing improved blood lipid profile also exhibited significant differences in their lifestyle habits after the 3-month intervention, with an increase in daily intake of vegetable and dairy products, as well as weekly exercise time (*P < *0.05). Moreover, two blood lipid metabolites (namely, acetyl-carnitine and free carnitine) significantly increased after probiotic supplementation cholesterol (*P < *0.05). In addition, probiotic-driven mitigation of hyperlipidemic symptoms were accompanied by increases in beneficial bacteria like Bifidobacterium animalis subsp. *lactis* and Lactiplantibacillus plantarum in patients’ fecal microbiota. These results supported that mixed probiotic application could regulate host gut microbiota balance, lipid metabolism, and lifestyle habits, through which hyperlipidemic symptoms could be alleviated. The findings of this study urge further research and development of probiotics into nutraceuticals for managing hyperlipidemia.

**IMPORTANCE** The human gut microbiota have a potential effect on the lipid metabolism and are closely related to the disease hyperlipidemia. Our trial has demonstrated that 3-month intake of a mixed probiotic formulation alleviates hyperlipidemic symptoms, possibly by modulation of gut microbes and host lipid metabolism. The findings of the present study provide new insights into the treatment of hyperlipidemia, mechanisms of novel therapeutic strategies, and application of probiotics-based therapy.

## INTRODUCTION

The rapid development of the world economy has dramatically changed people's living standards and diet structure, and the fast-food diet structure has also led to an obvious increase in the proportion of high-fat foods in the daily diet (1). A long-term high-fat diet will not only disrupt the balance of lipid metabolism in the body and cause metabolic disorders (2), but also lead to chronic diseases, such as hyperlipidemia (3), type 2 diabetes (4), hypertension (5), and obesity (6). Apart from being one of the most common metabolic disorders caused by a high-calorie diet, hyperlipidemia is also an important contributing factor in cardiovascular disease (7). The main therapeutic drugs for hyperlipidemia are statins, such as fluvastatin, atorvastatin, and rosuvastatin (8). However, it is worth noting that long-term use of statins can cause certain side effects to the body, and the drugs are relatively costly. Therefore, it is of interest to find alternative strategies for hyperlipidemia.

Several studies have found a link between hyperlipidemia and human gut microbiota, for example, significant differences in the gut microbiota profile between hyperlipidemic and healthy individuals (including animals) (9). It is also thought that alterations in the gut microbiome are an important cause promoting the onset and progression of hyperlipidemia (10). Long-term intake of a high-fat diet not only disrupts gut homeostasis, leading to gut microbial dysbiosis (11), but potentially damages the gastrointestinal barrier and permeability (12). Moreover, a long-term high-fat diet may lead to the reduction in both the ratio of gut Firmicutes to Bacteroidetes and the abundance of fatty acid-producing bacteria, such as *Bacteroides*, *Lactiplantibacillus*, and *Blautia* (13). In fact, the host gut microbiota are closely associated with some liver diseases, implicating the existence of an enterohepatic axis, which influences hepatic (fat) metabolism (14, 15). The liver is an important site for fat synthesis; however, liver cells can synthesize but not store the fat. Therefore, the synthesized fat is transported into the bloodstream after combining with apolipoproteins and cholesterols in the form of very low-density lipoproteins (16). The small intestine is another important site of lipid synthesis, and it is anticipated that the resident microbiota could play a critical role in the synthesis and transport of body fat.

Wang et al. found that feeding animals with high-fat diets disrupted both the host’s antioxidant capacity and gut microbiota balance, ultimately leading to the exacerbation of hyperlipidemic symptoms (17). Sun et al. found that a high-sucrose diet drastically shifted the gut microbiota in rats, characterized by an increase in the ratio of Bacteroidetes/Firmicutes, decrease in alpha diversity, and changes in diurnal oscillations, suggesting that a high-sucrose diet could reduce the level of blood fat via modulating the gut microbiota community (18). Ke et al. discovered that symbiotic intervention can reverse high fat diet (HFD)-induced changes in body weight gain, cholesterol, triglycerides, and microbial populations in mice through metabolic pathways involved in carbohydrate, amino acid, and energy metabolism (19). Yan et al. found that probiotics reversed the intestinal imbalance caused by hyperlipidemia by increasing the abundance of short-chain fatty acids-producing bacteria and thus the total amount of colonic short-chain fatty acids (13). In addition, supplementing probiotics-based products significantly reduced the weight gain and visceral obesity in hyperlipidemic mice and attenuated dyslipidemia via improving oxidative stress and chronic inflammation (20). Some *Lactiplantibacillus* spp. can regulate bile acid metabolism by downregulating farnesoid X receptor (FXR) genes and reduce exogenous cholesterol absorption by regulating Niemann-Pick C1-Like 1 (NPC1L1) (21). Provided the close association between the gut microbiota and hyperlipidemia, they can be regarded as a potential target for hyperlipidemia treatment. A growing body of literature shows that probiotics can modulate and even restore a healthier gut microbiota from disease-associated microbial dysbiosis states; thus, it is worth further investigating the clinical effects of probiotic application in managing hyperlipidemia.

This study hypothesized that probiotics could regulate the gut microbiota balance in hyperlipidemic patients and improve hyperlipidemic symptoms via modulating the host lipid metabolism. In this 3-month intervention trial, a total of 56 hyperlipidemic patients were recruited and randomized into the placebo and probiotic (receiving a mixed probiotic formulation) groups. The fecal microbiome and serum lipids of these patients were analyzed using metagenomics and liquid chromatography–mass spectrometry (LC-MS), respectively. Meanwhile, changes in patients’ physiological and biochemical parameters were recorded. This study confirmed the beneficial effect of Probio-X in managing hyperlipidemia.

## RESULTS

### Probio-X improved blood lipid indexes in a hyperlipidemia population.

Body weight, height, and work employment were collected and analyzed (Table 1). No significant difference was observed in the gender, ethnicity, age, height, weight, BMI, work status, marital status, and education level between probiotic and placebo groups (*P > *0.05 in all cases).

**TABLE 1:** Information of patients

Patients’ information	Groups		Chi-square test	
	Placebo (*n* = 27)	Probiotic (*n* = 29)	X^2^/*t*	*P*
Gender (male/female)	14/13	12/17	0.617	0.432
Ethnicity (Han/other)	20/7	21/8	0.020	0.889
Age (mean ± SD, yrs)	48.67 ± 11.79	51.17 ± 10.14	0.855	0.397
Ht (mean ± SD, m)	1.68 ± 0.85	1.63 ± 0.65	1.137	0.261
Wt (mean ± SD, kg)	76.30 ± 18.02	70.91 ± 13.76	0.917	0.363
BMI (mean ± SD, kg/m^2^)	27.09 ± 5.79	26.44 ± 4.16	0.507	0.614
Working status (full-time employment/other)	19/8	20/9	0.013	0.909
Marital status (married/other)	25/2	27/2	0.006	0.941
Education level (bachelor's degree or above/other)	20/7	20/9	0.179	0.672

**FIG 1:**
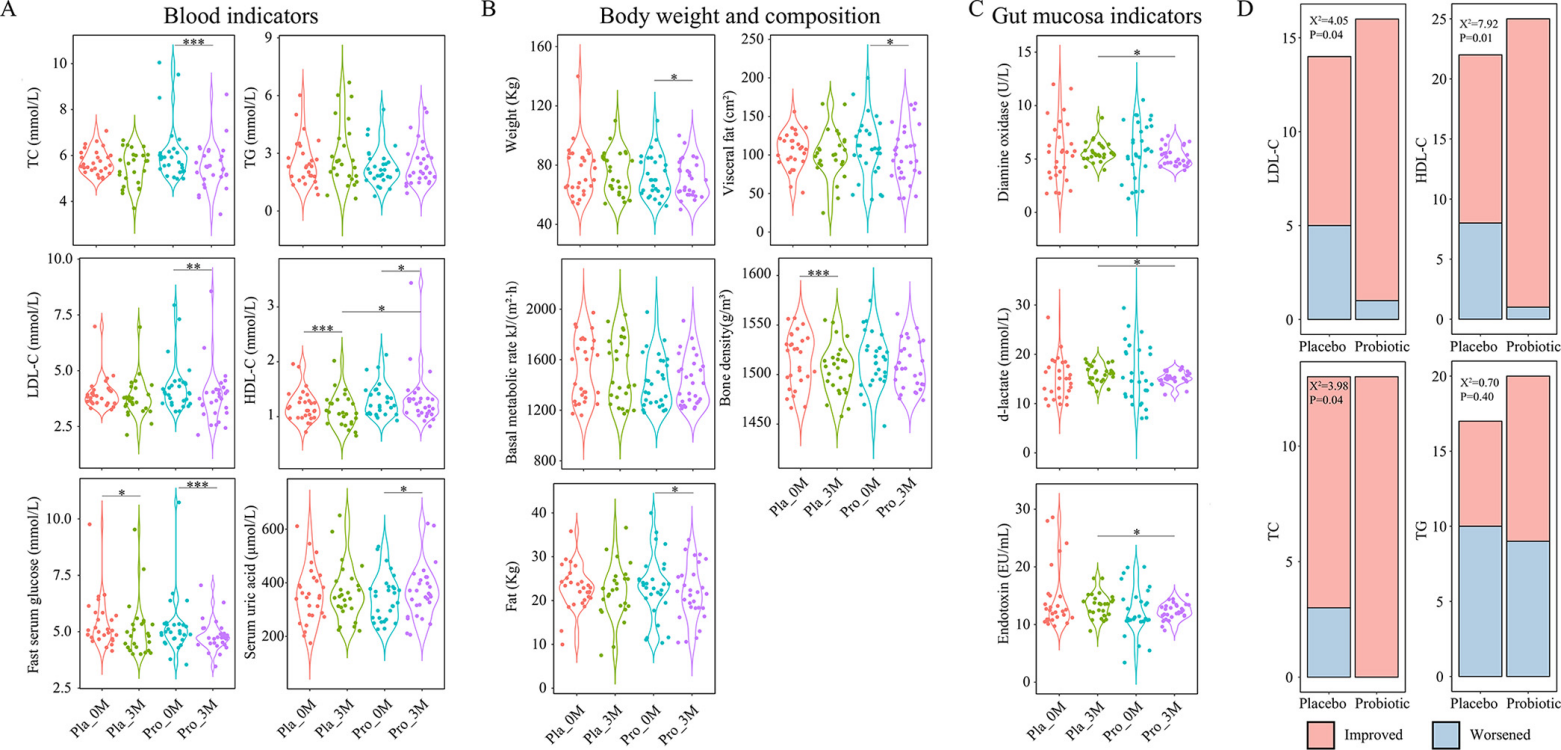
Probio-X improved blood lipid indexes in hyperlipidemia patients. (A) Comparative analysis of (A) blood indicators; (B) body weight and composition; (C) gut mucosa indicators. (D) Number of patients that were responsive toward probiotic treatment, evaluated by changes in four key blood lipid indicators. Chi-square test was performed to evaluate the overall difference between two groups. LDL-C, low-density lipoprotein; TC, total cholesterol; HDL-C, high-density lipoprotein; TG, triglyceride; *, *P < *0.05; **, *P < *0.01; ***, *P < *0.001 (*t* test). Pla_0M and Pla_3M indicate before and 3 months after the placebo intervention, respectively, and Pro_0M and Pro_3M before and 3 months after the probiotic intervention, respectively.

There were no significant differences in all blood biochemistry between the placebo and probiotic groups at baseline. The blood analysis of hyperlipidemic patients showed that the total cholesterol (TC) and low-density lipoprotein (LDL-C) levels decreased significantly after probiotic consumption (paired *t* test, *P < *0.05; Fig. 1A), while the high-density lipoprotein (HDL-C) levels increased significantly (paired *t* test, *P < *0.05). In contrast, in the placebo group, only blood HDL-C levels (paired *t* test, *P > *0.05), but not TC, triglyceride (TG), and LDL-C levels (paired *t* test, *P < *0.05), significantly decreased after the 3-month intervention period (*P < *0.05). However, both groups showed a significant decrease in fasting glucose (paired *t* test, *P < *0.05). The consumption of Probio-X not only significantly reduced the body weight, visceral fat, and blood fat of patients, but also effectively prevented loss in their bone mineral density (paired *t* test, *P > *0.05; Fig. 1B). The analysis of gut mucosa indicators (Fig. 1C) revealed no significant change in diamine oxidase, d-lactate, and endotoxin in both groups of patients (paired *t* test, *P > *0.05), though it is worth noting that the probiotic group had significantly lower values in the aforementioned indicators (unpaired *t* test, *P < *0.05).

We further analyzed changes in the four main blood lipid indicators in the placebo and probiotic groups (Fig. 1D), and our results revealed that significantly more patients in the probiotic group showed improvements in TC, LDL-C, and HDL-C than the placebo group (chi-square test, *P < *0.05), while no significant difference was found in the number of patients showing improvement in TG (chi-square test, *P > *0.05). These results demonstrated that the consumption of Probio-X could significantly improve some major clinical indicators related to hyperlipidemia.

### Probio-X improved dietary and exercise habits in hyperlipidemic patients.

**FIG 2:**
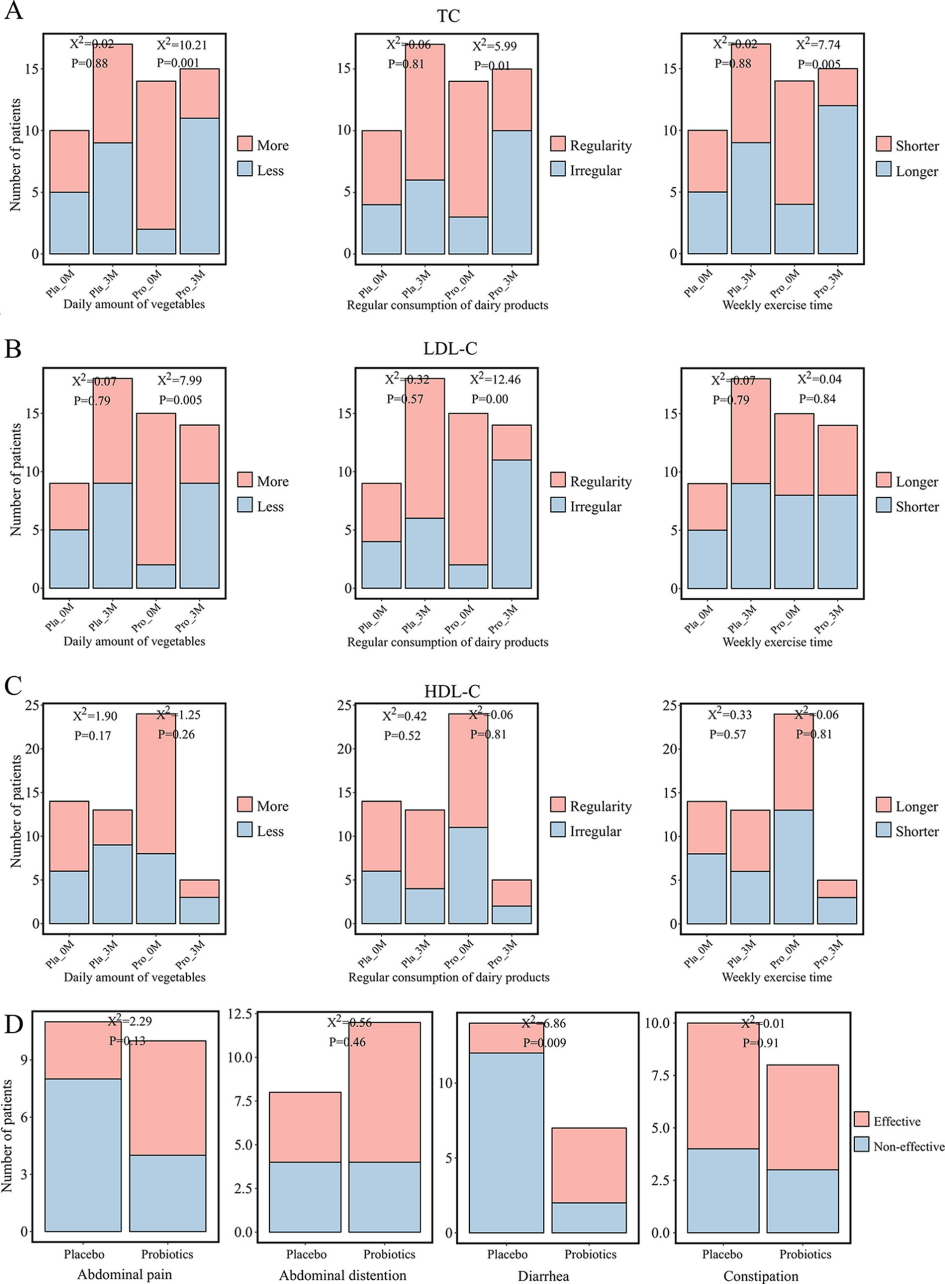
Probio-X improved dietary and exercise habits and gastrointestinal wellness in hyperlipidemia patients. Comparative analysis of habits of vegetables/dairy product consumption and weekly exercise in (A) patients with normal total cholesterol (TC); (B) low-density lipoprotein (LDL-C) populations; and (C) high-density lipoprotein (HDL-C) remission populations. (D) Comparative analysis of patients’ feelings of gastrointestinal wellness. Pla_0M and Pla_3M indicate before and 3 months after the placebo intervention, respectively, and Pro_0M and Pro_3M before and 3 months after the probiotic intervention, respectively.

A self-administered questionnaire was applied to record the dietary and exercise habits of the hyperlipidemic patients during the trial intervention. We thus analyzed changes in dietary and exercise habits in patients showing improvements in TC, LDL-C, and HDL-C. Interestingly, patients in the probiotic group that showed a significant decrease in TC (Fig. 2A) also increased their daily vegetable and dairy product consumption, as well as time of weekly exercise, compared to the preintervention period (chi-square test, *P < *0.05), while those in the placebo group did not show any significant changes in these aspects after the intervention (chi-square test, *P > *0.05). Similarly, patients in the probiotic group that had lowered LDL-C levels (Fig. 2B) increased daily vegetable and dairy product consumption (chi-square test, *P < *0.05), though no significant change was observed in their weekly exercise time compared to preintervention (chi-square test, *P > *0.05). Such changes were not observed in the placebo group (chi-square test, *P > *0.05). In contrast, patients from both the probiotic and placebo groups showing improvement in HDL-C (Fig. 2C) did not exhibit obvious changes in their dietary and exercise habits (chi-square test, *P > *0.05). Meanwhile, the patients’ feeling of gastrointestinal wellness was analyzed (Fig. 2D). Significantly less diarrhea frequency was observed in subjects given Probio-X compared with those receiving placebo material (chi-square test, *P < *0.05), while there was no significant difference in the occurrences of abdominal pain, bloating, and constipation between the two groups (chi-square test, *P > *0.05). These results together showed that the improvement in major blood lipid indexes was accompanied by healthier lifestyle changes.

### Changes in patients’ fecal microbiota after Probio-X intervention.

**FIG 3:**
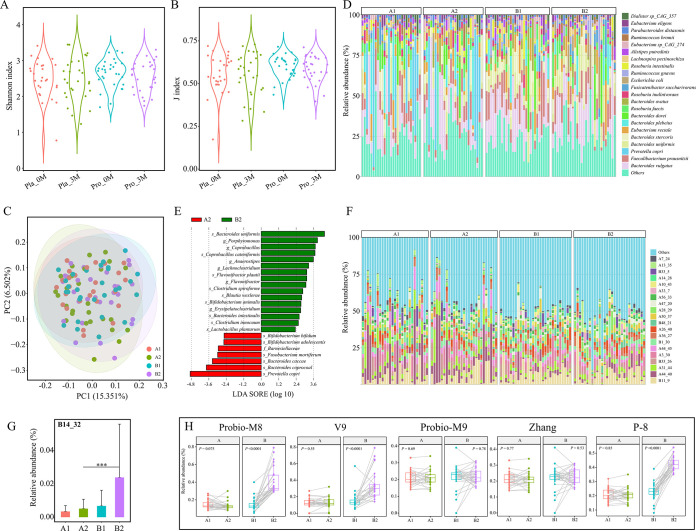
Changes in fecal microbiota after Probio-X intervention. (A) Shannon index. (B) J index. (C) Relative abundance of dominant species. (D) Principal coordinate analysis (Bray-Curtis distance). (E) Linear discriminant analysis effect size (LEfSe) analysis. Distribution of (F) dominant metagenome-assembled genomes (MAGs) in each subject; (G and H) the MAG, B14_32, and the applied probiotic strains (Probio-M8, Bifidobacterium lactis Probio-M8; V9, Bifidobacterium lactis V9; Probio-M9, *Lactiplantibacillus rhamnosus* Probio-M9; Zhang, *Lactiplantibacillus casei* Zhang; P-8, *Lactiplantibacillus plantarum* P-8) in different sample groups. Pla_0M and Pla_3M indicate before and 3 months after the placebo intervention, respectively, and Pro_0M and Pro_3M before and 3 months after the probiotic intervention, respectively. A1 represents Pla_0M, A2 represents Pla_3M, B1 represents Pro_0M, and B2 represents Pro_3M.

Although numerous studies in recent years have observed a strong association between the gut microbiota and onset/progression of hyperlipidemia, our microbial alpha diversity analysis (Shannon and J indexes) of subjects’ fecal microbiota revealed no significant intra-/intergroup differences (paired Wilcoxon test, *P > *0.05; unpaired Wilcoxon test, *P > *0.05, respectively; Fig. 3A and B), suggesting that probiotic application did not significantly change the alpha diversity of subjects. Then, a principal coordinate analysis (Bray-Curtis distance, Fig. 3C) was performed to evaluate differences between groups/subgroups. On the principal coordinate score plot, symbols of different groups/subgroups showed a high degree of overlap, indicating no significant differences in the overall intra-/intergroup microbiota structure. The fecal microbiota composition was similar to those reported in previous studies (22), and the major species were mostly common microbes residing in the human intestine (Fig. 3D). Differential microbes between probiotic and placebo groups after the 3-month intervention were detected using linear discriminant analysis effect size (LEfSe), and a total of 22 differential species were found (Fig. 3E). Notably, both Bifidobacterium animalis and Lactiplantibacillus plantarum were significantly enriched after probiotic intervention.

This study also analyzed the profile of metagenome-assembly genomes (MAGs) in the fecal microbiota (Fig. 3F). Some differential MAGs were detected, including B14_32 (corresponding to Bifidobacterium animalis subsp. *lactis*), that were significantly more enriched in the probiotic group than the placebo group after the 3-month intervention (Fig. 3G). Finally, by mapping the reads to the genomes of probiotics, the relative abundance of all five applied strains of probiotics could be identified from patients’ gut microbiome. For the placebo group, the relative abundances of all five strains did not show obvious changes after the 3-month treatment (paired Wilcoxon test, *P > *0.05; Fig. 3H), while significant increases were observed in three of the strains (Bifidobacterium animalis Probio-M8, Bifidobacterium animalis V9, and *Lactiplantibacillus plantarum* P-8) after 3 months of probiotic mix intervention (paired Wilcoxon test, *P < *0.05; Fig. 3H).

### Changes in patients’ serum lipid profile after Probio-X intervention.

**FIG 4:**
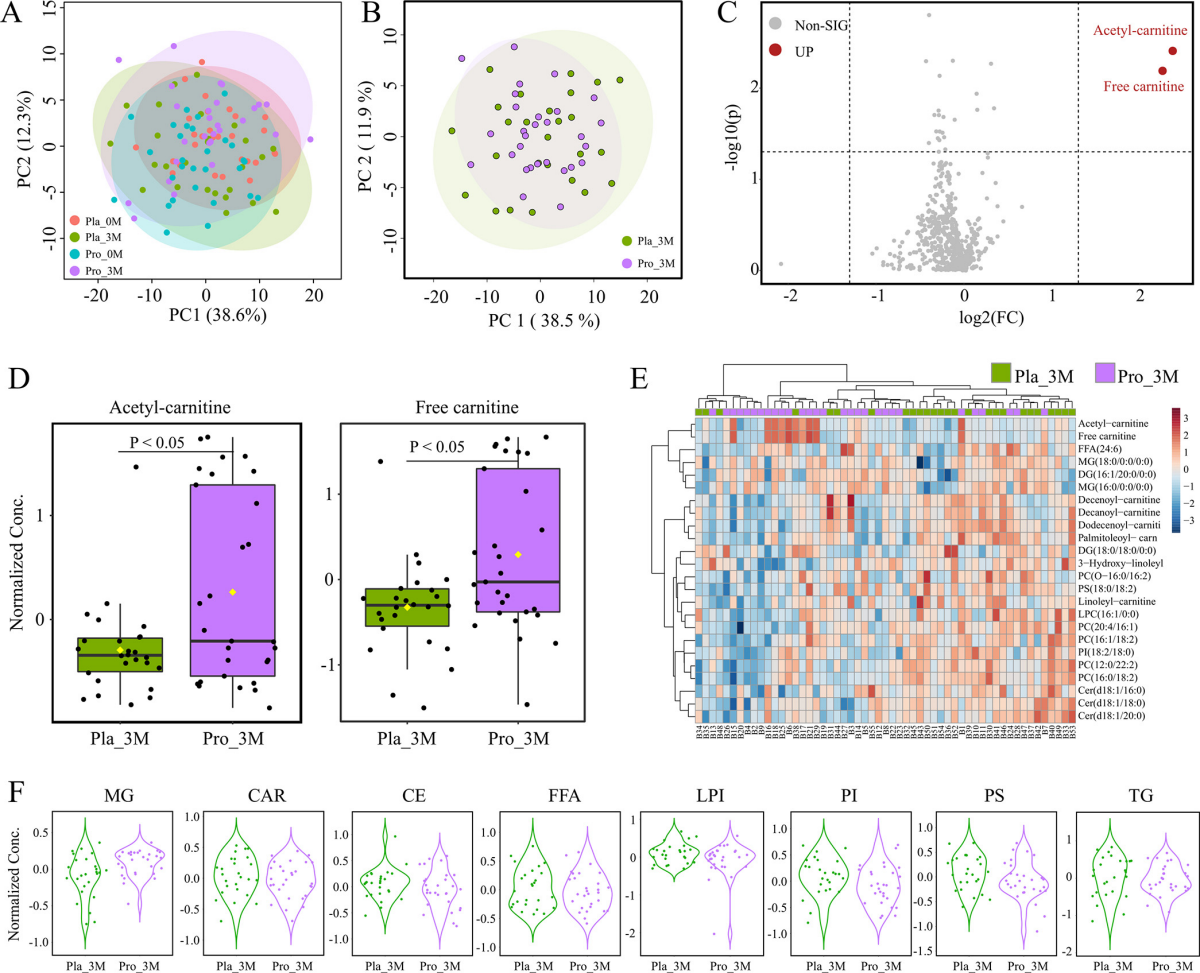
Changes in serum lipid profile after Probio-X intervention. (A and B) Principal-component analysis of serum lipid metabolomes of subjects. Each dot represents the serum lipid metabolome of each subject. (C) Volcano map showing detected serum lipid metabolites. Non-SIG represented metabolites without significant increase after probiotic intervention, UP represented metabolites with significant increase after probiotic intervention. Significantly increased metabolites are shown in red. (D) Relative abundance heatmap of differential metabolites. The color scale represents the relative abundance of metabolites. (E) Box plots showing the normalized amounts of acetyl-carnitine and free carnitine after probiotic intervention. (F) Violin plots showing differences in various lipid metabolites after probiotic intervention. G, monoglyceride; CAR, acyl carnitine; CE, cholesterol lipids; FFA, free fatty acids; LPI, lysophosphatidylinositol; PI, phosphatidylinositol; PS, phosphatidyl serine. Pla_0M and Pla_3M indicate before and 3 months after the placebo intervention, respectively, and Pro_0M and Pro_3M before and 3 months after the probiotic intervention, respectively.

It is known that gut microbiota interacts with the host metabolism and influences the blood metabolome. Therefore, patients’ lipid blood metabolic profile was analyzed by principal-component analysis (Fig. 4). However, symbols representing the four subgroups (placebo and probiotic groups at baseline and after 3-month intervention) largely overlapped on the principal-component analysis score plot, indicating that the probiotic treatment did not cause significant changes in the overall blood lipid metabolome structure (Fig. 4A and B). Then, volcano plot analysis was performed to look for differential metabolites between placebo and probiotics groups after the intervention, which identified only two significantly increased differential metabolites, acetyl-carnitine and free carnitine after probiotic intervention (fold change [FC] > 2, *P < *0.05; Fig. 4C and D). There was, however, a general decrease in other differential metabolites identified after postprobiotic intervention (identified only based on paired Wilcoxon test, *P < *0.05; Fig. 4E). A finer analysis of the blood lipid metabolites based on class grouping (into eight categories: MG, monoglyceride; CAR, acyl carnitine; CE, cholesterol lipids; FFA, free fatty acids; LPI, lysophosphatidylinositol; PI, phosphatidylinositol; PS, phosphatidyl serine, TG, triglyceride; Fig. 4F) revealed no significant difference in all metabolite categories between probiotic and placebo groups after the intervention (unpaired Wilcoxon test, *P > *0.05).

### Changes in predicted gut metabolites after Probio-X intervention.

**FIG 5:**
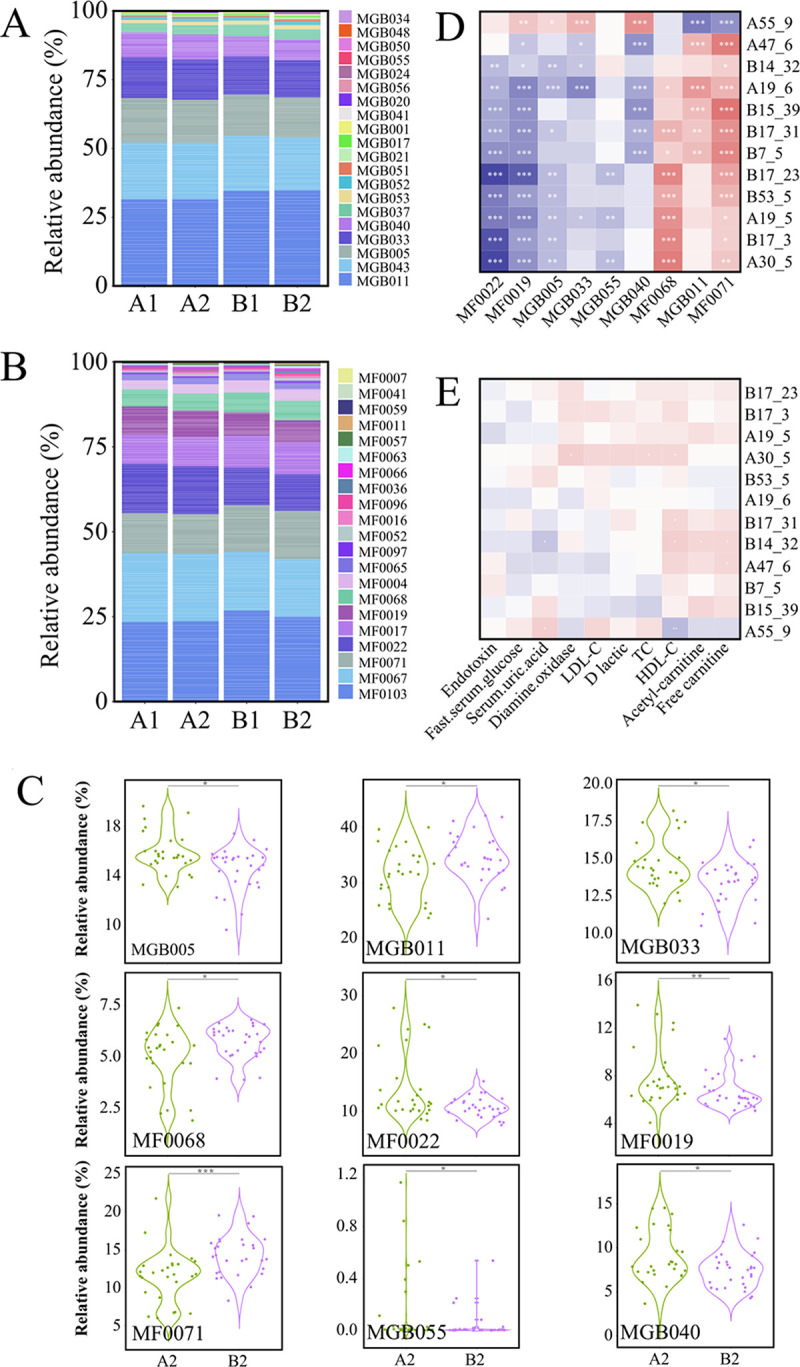
Predicted gut metabolites. (A and B) Relative abundance of predicted gut metabolites. (C) Violin plots showing differential gut metabolites. Correlation heatmap of (D) metagenome-assembly genome (MAGs) and gut metabolites; (E) MAGs and blood indicators. Red indicates positive correlation, blue indicates negative correlation, and the color intensity is the strength of correlation. *, *P < *0.05; **, *P < *0.01; ***, *P < *0.001.

The probiotic treatment did not seem to drastically modulated patients’ fecal microbiota structure and composition; thus, we further investigated the mechanisms of regulation of hyperlipidemia by analyzing probiotics-driven changes in predicted gut metabolites from the MAGs. A total of 41 gut metabolites were predicted across 337 MAGs (selection criteria: integrity greater than 80, contamination less than 10, and relative abundance greater than 1%; data not shown). The gut metabolite, MGB043, was common to the majority of MAGs, while MGB034, MGB055, MGB056, and MF0059 were predicted to be present exclusively in only one MAG. A total of 20 gut metabolites were predicted from the MGB database, including the major metabolites MGB011 and MGB043 (Fig. 5A), while a total of 21 gut metabolites were predicted from the MF database (MF0103 and MF0067 as the major metabolites; Fig. 5B). Forty-one significantly differential metabolites between the probiotic and placebo groups were detected after the intervention (Fig. 5C); nine were significantly enriched (e.g., cortisol degradation, glycolysis, and pentose phosphate pathway; unpaired Wilcoxon test, *P < *0.05 in each case) or deprived (e.g., tryptophan synthesis, quinolinic acid degradation, and galacturonate degradation I et al.; unpaired Wilcoxon test, *P < *0.05 in each case) after the probiotic treatment.

Correlation analysis revealed significant positive correlations between three predicted differential gut metabolites (cortisol degradation, glycolysis, and pentose phosphate pathway) and almost all differential MAGs (Spearman's test, *P < *0.05; Fig. 5D), while other differential gut metabolites showed significantly negative correlations with almost all differential MAGs (Spearman 's test, *P < *0.05; Fig. 5D), except Paraprevotella clara, which showed an opposite trend. Moreover, acetyl-carnitine and free carnitine correlated significantly and positively with two MAGs, Bifidobacterium animalis and Blautia obeum (Spearman's test, *P < *0.05; Fig. 5E); HDL-C was significantly and negatively correlated with Paraprevotella clara, but was positively correlated with Bifidobacterium animalis, Veillonella parvula, and Erysipelatoclostridium ramosum (Spearman's test, *P < *0.05 in all cases; Fig. 5E). Serum uric acid showed a significant positive correlation with Paraprevotella clara, but negatively correlated with Bifidobacterium animalis (Spearman's test, *P < *0.05; Fig. 5E).

## DISCUSSION

Gut microbiota are associated with the host metabolism and may influence the onset and development of hyperlipidemia (23). Probiotics have been shown to lower blood TC, LDL-C, and TG, alleviating metabolic syndrome in hyperlipidemic rats (21). This study analyzed the effects of a mixed probiotic formulation on hyperlipidemia, with focus on changes in patients’ gut microbiota and their metabolic potential.

Our data supported that the intake of the probiotic mix could effectively reduce the serum levels of TC and LDL-C, while increasing serum HDL-C levels, in patients with hyperlipidemia. Such results supported that probiotic application could regulate hosts’ lipid metabolism. There are different subtypes of dyslipidemia (e.g., high cholesterol and/or high triglycerides), which would require different treatment approaches. For example, Yan et al. found that oryzanol could reduce the TC of serum in rats (24), while Liu et al. found that Tartary buckwheat protein could significantly reduce LDL-C levels and increase HDL-C levels in the sera of hyperlipidemic rats (25). Although these functional products could reduce some of the blood lipid indices in hyperlipidemic rats, only the Probio-X applied to this could effectively modulate all three important blood lipid indexes, TC, LDL-C, and HDL-C, in hyperlipidemic subjects, suggesting that probiotics are potential functional products for alleviating hyperlipidemia.

The results of the self-administered questionnaire revealed interesting and desirable lifestyle habit changes (such as increased daily vegetable consumption, regular dairy consumption, and increased weekly exercise time) in hyperlipidemic patients that showed significant improvement in blood lipid indexes (TC, LDL-C, and HDL-C) after probiotic consumption. Indeed, there was a strong association between the desirable changes in patients’ lifestyle habits and lowering of these indexes. Wastyk et al. believe that diet and gut microbiota are interacting bidirectionally. In other words, diet regulates the gut microbiota, and vice versa (26). A previous study also demonstrated an influential role of intake of vegetables/dairy products and exercise in regulating serum levels of TC, LDL-C, and HDL-C (27). Moreover, apart from lower hyperlipidemia, the results of this study also found that taking Probio-X could significantly alleviate diarrhea. Similarly, McFarland et al. confirmed the effectiveness of probiotics in treating diarrhea (28). The meta-analysis of Lu et al. observed a significantly reduced total diarrhea rate in cancer patients when probiotics were used in conjunction with the conventional treatment (29). Numerous studies consistently showed that probiotic intake could alleviate diarrhea, which was likely achieved by regulating the host's gut microbial balance (30). Thus, the Probio-X applied in this study could alleviate hyperlipidemia and diarrhea.

Although a large body of study has supported that the gut microbiota serve as an important mediator of lipid metabolism, our study did not observe drastic probiotic-driven changes in the fecal microbiota structure and composition, which could be related to the high complexity of the human gut microbiota. Similar observation was made by Liu et al., where the authors investigated effects of probiotics on the gut microbiome of asthmatic patients (31). Differential species were only identified between probiotic and placebo groups at a fine taxonomic level. For example, we noted significant increases in the species Bifidobacterium animalis subsp. *lactis* and Lactiplantibacillus plantarum postprobiotic intervention. The increases in some of the applied strains in the mixed probiotic preparation (including Probio-M8, V9, and P-8) in the fecal microbiome of the probiotic group were confirmed by MAG analysis. A previous investigation showed that intestinal Bifidobacterium animalis subsp. *lactis* could improve hyperlipidemia by regulating bile acid metabolism through downregulating FXR genes while reducing exogenous cholesterol absorption through modulating the NPC1L1 gene (21). Chen found that *Lactiplantibacillus plantarum* could reduce serum levels of TG, TC, and LDL-C, which in turn inhibited excessive accumulation of liver fat (32). Our results supported that the Probio-X-driven hyperlipidemic symptom improvement was accompanied by modulating a specific portion of the intestinal microbiota.

The blood lipid metabolome of subjects did not change significantly regardless of probiotic intervention, but further analysis of lipid metabolites identified two differential metabolites, i.e., acetyl-carnitine and free carnitine, that were significantly increased after the mixed probiotic intervention. The human body, as an extremely complex microecosystem, has a strong capacity to maintain organismal stability by self-regulation, which may be one of the reasons for the nonsignificant change in the overall blood metabolome and low number of differential metabolites. However, it is worth noting that acetyl-carnitine plays an integral role in lipid metabolism by participating in the β-oxidation of long-chain fatty acids (33). Seccombe demonstrated that acetyl-carnitine could alleviate hepatic steatosis caused by a high-fat diet and reduce plasma levels of TC and TG (34). Additionally, free carnitine is beneficial in lowering blood lipids (35).

Our further analysis found that Bifidobacterium animalis subsp. *lactis* correlated positively with acetyl-carnitine and free carnitine, as well as the predicted gut metabolic pathway, representing γ-aminobutyric acid (GABA) synthesis. Since acetyl-carnitine and free carnitine are known to have high affinity with certain GABA uptake sites (36, 37), we speculate that Bifidobacterium animalis subsp. *lactis* may promote the transport of acetyl-carnitine and free carnitine through GABA synthesis, then elevate their serum levels and thus improve the blood lipid profile. In conclusion, our data support that administering Probio-X could improve hyperlipidemia by modulating gut microbes and their metabolites.

### Conclusions.

We performed a 3-month intervention trial and confirmed the beneficial effects of application of a probiotic mix in alleviating hyperlipidemia, at least in part, by regulating the blood lipid indexes (lowering serum TC, LDL-C, and HDL-C), lipid metabolism, and gut microbiota. Although insignificant changes were observed in the lipid metabolome and gut microbiota structure, some interesting fecal bacteria (e.g., Bifidobacterium animalis and *Lactiplantibacillus plantarum*) and blood metabolites (e.g., acetyl-carnitine and free carnitine) increased significantly after Probio-X intervention, suggesting the probiotic-driven treatment improvement of hyperlipidemic effects could be through modulation of these gut microbes and host lipid metabolism. Collectively, our study serves as an additional study supporting that probiotic administration is a promising approach in managing hyperlipidemia and improving public health.

## MATERIALS AND METHODS

### Probiotic administration.

Probio-X (3 × 10^10^ CFU/g) contains equal proportions of Lactiplantibacillus casei Zhang (*L. casei* Zhang), Bifidobacterium lactis V9 (*B. lactis* V9), Bifidobacterium lactis Probio-M8 (*B. lactis* Probio-M8), Lactiplantibacillus rhamnosus Probio-M9 (L. rhamnosus Probio-M9), and Lactiplantibacillus plantarum P-8 (*L. plantarum* P-8). *Lactiplantibacillus casei* Zhang was isolated from a sour horse milk product in Inner Mongolia; Bifidobacterium lactis V9 was isolated from the feces of healthy children; Bifidobacterium lactis Probio-M8 and *Lactiplantibacillus rhamnosus* Probio-M9 were isolated from breast milk samples of healthy mothers; and *Lactiplantibacillus plantarum* P-8 was isolated from a traditionally fermented dairy product in Inner Mongolia. *L. casei* Zhang, *B. lactis* V9, *B. lactis* Probio-M8, L. rhamnosus Probio-M9, and *L. plantarum* P-8 showed good tolerance to conditions simulating the gastrointestinal tract (such as in environments containing gastric acid, intestinal fluid, and bile) and abilities to improve the host gut microbiota structure and health.

### Trial design and subject recruitment.

This study was a 3-month randomized controlled double-blind trial. A total of 112 patients with hyperlipidemia were recruited. All patients were recruited by the Inner Mongolia People's Hospital and Inner Mongolia Agricultural University. The inclusion criteria were as follows: (i) male or female aged between 18 and 65 with diagnostic criteria for hyperlipidemia; and (ii) willingness to commit throughout the trial. In contrast, the exclusion criteria were as follows: (i) a history of major diseases, such as cancer, kidney disease, gastrointestinal diseases, mental illness, or type I diabetes; (ii) takin antibiotics, probiotics, prebiotics, postbiotics, and immunosuppressive agents within 1 month before the intervention or during the intervention; (iii) irregular eating habits during the intervention and follow-up period; and (iv) failure to collect stool and blood samples twice. Forty-three subjects were excluded after the first round of screening; sixty-nine patients were randomly assigned to probiotic (*n* = 35) and placebo (*n* = 34) groups. After being informed of the specific experimental guidelines and details, three patients withdrew from the trial; seven patients discontinued intervention for their consumption of antibiotics or yogurt; and three patients lacked stool samples. Thus, 29 and 27 subjects subsequently remained in the probiotic and placebo groups. Both participants and researchers were unaware of the group allocation throughout the trial. The two groups of patients received a daily dose of two grams of probiotic or placebo preparation, respectively. The placebo preparation comprised maltodextrin, which was identical to the probiotic preparation in packaging and in sensory aspects (e.g., taste and smell). The probiotic preparation contained the placebo material (excipient) plus probiotics (a total of 3 × 10^10^ CFU/g). No patients took any probiotics, antibiotics, or yogurt during the trial. Patients’ diet and lifestyle information were collected by a self-administered questionnaire for subsequent correlation analyses. Written, informed consent from legal guardians was obtained from each participant.

### Collection and processing of stool and blood samples.

The study was conducted for 3 months, and feces and blood were collected from all participants at days 0 and 90 after the start of the study. Stool samples were self-collected by each subject at the specified time point at home using a uniformly provided sterile stool sampler with DNA protection solution (Guangdong Longhai Biomedical Co., Ltd., Guangzhou, China). All patients were given prior training in the fecal collection procedure to prevent fecal contamination during the collection process. Collected samples were transported to the hospital at low temperature and were stored in a −80°C freezer before the next steps of sample processing for deep metagenomic sequencing.

All patients were asked to visit the hospital after stool sample collection, when blood samples (after a 12-h fast) were taken. All collected blood samples were stored in sterile centrifuge tubes, before centrifuging at 4°C for 15 min at 3,000 *g* for serum collection. Collected serum samples were stored at −80°C pending further analysis.

The study was approved by the Ethics Committee of the Inner Mongolia People's Hospital (no. 201811002) and was registered on the Chinese Clinical Trial Registry (http://www.chictr.org.cn/; registration number ChiCTR1900026469). Informed consent was obtained from all recruited subjects prior to the study.

### Analysis of biochemical indicators.

Blood samples were used for monitoring physiological and biochemical changes in the subjects. The analyzed physiological/biochemical parameters mainly focused on lipid metabolism, namely, LDL-C, TC, HDL-C, high-sensitivity C-reactive protein [hsCRP], fasting serum glucose, TG, serum uric acid, diamine oxidase, d-lactate, and endotoxin. All biochemical analysis was performed by the Inner Mongolia People's Hospital.

### Fecal metagenomics analysis.

**(i) DNA extraction.** After thawing the fecal samples, two grams of each fecal sample was weighed and used for metagenomic DNA extraction. The metagenomic DNA was extracted with the QIAamp DNA Stool minikit (Qiagen, Germany) according to the manufacturer's instructions. The purity and quality of the extracted DNA were ensured by a NanoDrop spectrophotometer (Thermo Scientific, USA) and agarose gel electrophoresis. All samples were stored in a −20°C freezer prior to further processing (38).

**(ii) Shotgun metagenomic sequencing.** One microgram DNA per sample was used for sequencing library preparation. Sequencing libraries were generated using NEBNext Ultra DNA Library Prep kit for Illumina (NEB, USA) following the manufacturer’s recommendations. Index codes were applied to attribute sequences of each sample. Shotgun sequencing was performed on the HiSeq 2500 platform (Illumina, CA, USA). Paired-end reads (151 bp in length) were generated.

**(iii) Bioinformatic analysis.** The reads were trimmed using FastQC (v 0.11.9) (https://github.com/s-andrews/FastQC) and were subsequently aligned to the human genome to remove host DNA sequences. The remaining high-quality reads were further analyzed. After removing the human and low-quality sequences, a total of 1258.36 Gb of high-quality sequence data was generated from all samples (average of 11.24 Gb per sample). J-Index and Shannon index were used to characterize the abundance of community microorganisms of subjects’ fecal microbiota (14).

The fecal microbiota composition was obtained by comparing high-quality reads with standard databases using Metaphlan 2, ver. 2.0 (39). High-quality reads were assembled into contigs using MegaHit, ver. 1.0 (40), and the obtained contigs were binned into high-quality scaffolds by MetaBAT2, ver. 2.12.1 (41), VAMB, ver. 1.0 (42), and Maxbin2, ver. 2.0 (43). Meanwhile, the obtained high-quality scaffolds were screened for metagenome-assembly genomes (MAGs) using the Das tool, v 1.1.2 (44). The quality of the obtained MAGs was assessed by CheckM, ver. 1.0.18 (45) to screen for high-quality MAGs and gene set construction (completeness > 80% and contamination < 5%). High-quality MAGs were clustered by dRep, ver. 2.2.4 (46) to define species-level genome bins (SGBs). The taxonomy of each MAG was annotated against the National Center for Biotechnology Information (NCBI) nonredundant Nucleotide Sequence Database (NT) using BLASTn. The Kyoto Encyclopedia of Genes and Genomes (KEGG) was annotated using eggnog-mapper. Metabolic profiles of the fecal microbes were predicted using MelonnPan (https://huttenhower.sph.harvard.edu/melonnpan/).

**(iv) Nucleotide sequence accession numbers.** The sequence dataset generate in this study has been deposited in the NCBI Sequence Read Archive (SRA) database (PRJNA946655).

### Serum metabolomics analysis.

**(i) Sample preparation and extraction.** Serum samples were thawed, vortexed for 10 s, and centrifuged for 5 min (4°C and 3,000 rpm). Then, 50 μL of each sample was accurately pipetted into a prelabeled centrifuge tube containing 1 mL of lipid extract (including the standard mixture). The mixture was vortexed for 2 min, sonicated for 5 min, before adding 500 μL of distilled water, vortexed for 1 min, and centrifuged for 10 min (4°C and 1,2000 rpm). Then, 500 μL of the supernatant was accurately pipetted into a new prelabeled centrifuge tube, which was then concentrated and reconstituted into a final volume of 100 μL with acetonitrile containing 0.04% acetic acid (i.e., the mobile phase B for LC- tandem mass spectrometry, MS/MS, analysis).

**(ii) Ultrahigh performance liquid chromatography-quadrupole time-of-flight mass spectrometry (UPLC-Q-TOF MS).** Serum lipid profile was mainly performed by UPLC Shim-pack UFLC SHIMADZU CBM30A, SHIMADZU, Kyot liquid phase were: the column (Water ACQUITY UPLC HSS T3 C18 1.8 μm, 2.1 mm*100mm); mobile phases, ultrapure water (0.04% acetic acid) for phase A and acetonitrile (0.04% acetic acid) for phase B; flow rate of 0.4 mL/min; column temperature of 40°C; injection volume of 5 μL. The MS conditions were as follows: electrospray ionization (ESI) temperature of 550°C; MS voltages of 5,500 V (positive) and −4,500 V (negative); ion source gas I of 55 lb/in^2^, gas II of 60 lb/in^2^, curtain gas of 25 lb/in^2^; and collision-activated dissociation parameters set to high (47). Qualitative analysis was performed using a self-built metware database, MWDB, identifying metabolites based on retention time, ion pair information, and secondary spectrum data of the detected substances.

### Statistical analysis and data visualization.

All statistical analyses were performed using the R software (v 4.0.3), and data were expressed as mean ± SD. Wilcoxon test and *t* test were used to evaluate differences in blood indicators, fecal microbiome, and lipid metabolites between groups/subgroups. The chi-square test was used to calculate the difference between counts. The R package vegan was used for alpha and beta diversity analysis. The R package Psych was used for Spearman’s correlation analysis. Principal coordinate analysis (Bray-Curtis distance) and principal-component analysis were performed with R. LEfSe analysis was performed with R. Data were visualized with R software.

## References

[1] Zhu Z, Lin Z, Jiang H, Jiang Y, Zhao M, Liu X. 2017. Hypolipidemic effect of Youcha in hyperlipidemia rats induced by high-fat diet. Food Funct 8:1680–1687. doi:10.1039/C7FO00089H. 28379241

[2] Zhou X, Li Z, Qi M, Zhao P, Duan Y, Yang G, Yuan L. 2020. Brown adipose tissue-derived exosomes mitigate the metabolic syndrome in high fat diet mice. Theranostics 10:8197–8210. doi:10.7150/thno.43968. 32724466 PMC7381731

[3] Feng K, Zhu X, Chen T, Peng B, Lu M, Zheng H, Huang Q, Ho CT, Chen Y, Cao Y. 2019. Prevention of obesity and hyperlipidemia by heptamethoxyflavone in high-fat diet-induced rats. J Agric Food Chem 67:2476–2489. doi:10.1021/acs.jafc.8b05632. 30740980

[4] Guo XX, Wang Y, Wang K, Ji BP, Zhou F. 2018. Stability of a type 2 diabetes rat model induced by high-fat diet feeding with low-dose streptozotocin injection. J Zhejiang Univ Sci B 19:559–569. doi:10.1631/jzus.B1700254. 29971994 PMC6052359

[5] Liu Y, Gao Y, Ma F, Sun M, Mu G, Tuo Y. 2020. The ameliorative effect of *Lactobacillus plantarum* Y44 oral administration on inflammation and lipid metabolism in obese mice fed with a high fat diet. Food Funct 11:5024–5039. doi:10.1039/D0FO00439A. 32530448

[6] Hsu CN, Hou CY, Chan JYH, Lee CT, Tain YL. 2019. Hypertension programmed by perinatal high-fat diet: effect of maternal gut microbiota-targeted therapy. Nutrients 11:2908. doi:10.3390/nu11122908. 31810197 PMC6950030

[7] Alloubani A, Nimer R, Samara R. 2021. Relationship between hyperlipidemia, cardiovascular disease and stroke: a systematic review. Curr Cardiol Rev 17:e051121189015. doi:10.2174/1573403X16999201210200342. 33305711 PMC8950504

[8] Li C, Liu H, Yang J, Mu J, Wang R, Zhao X. 2020. Effect of soybean milk fermented with *Lactobacillus plantarum* HFY01 isolated from yak yogurt on weight loss and lipid reduction in mice with obesity induced by a high-fat diet. RSC Adv 10:34276–34289. doi:10.1039/D0RA06977A. 35519026 PMC9056763

[9] Li H, Liu B, Song J, An Z, Zeng X, Li J, Jiang J, Xie L, Wu W. 2019. Characteristics of gut microbiota in patients with hypertension and/or hyperlipidemia: a cross-sectional study on rural residents in Xinxiang County, Henan Province. Microorganisms 7:399. doi:10.3390/microorganisms7100399. 31561625 PMC6843550

[10] Li Y, Ma Q, Wang J, Li P, Cheng L, An Y, Duan Y, Dai H, Wang T, Zhao B. 2020. Relationship between hyperlipidemia and the gut microbiome of rats, characterized using high-throughput sequencing. J Traditional Chinese Medical Sciences 7:154–161. doi:10.1016/j.jtcms.2020.04.006.

[11] He C, Cheng D, Peng C, Li Y, Zhu Y, Lu N. 2018. High-fat diet induces dysbiosis of gastric microbiota prior to gut microbiota in association with metabolic disorders in mice. Front Microbiol 9:639. doi:10.3389/fmicb.2018.00639. 29686654 PMC5900050

[12] Zhou D, Pan Q, Xin FZ, Zhang RN, He CX, Chen GY, Liu C, Chen YW, Fan JG. 2017. Sodium butyrate attenuates high-fat diet-induced steatohepatitis in mice by improving gut microbiota and gastrointestinal barrier. World J Gastroenterol 23:60–75. doi:10.3748/wjg.v23.i1.60. 28104981 PMC5221287

[13] Yan J, Xue Q, Chen W, Wang K, Peng D, Jiang J, Li P, Du B. 2022. Probiotic-fermented rice buckwheat alleviates high-fat diet-induced hyperlipidemia in mice by suppressing lipid accumulation and modulating gut microbiota. Food Res Int 155:111125. doi:10.1016/j.foodres.2022.111125. 35400410

[14] He Q, Yang C, Kang X, Chen Y, Zhang T, Zhang H, Kwok LY. 2022. Intake of *Bifidobacterium lactis* Probio-M8 fermented milk protects against alcoholic liver disease. J Dairy Sci 105:2908–2921. doi:10.3168/jds.2021-21265. 35086715

[15] Tilg H, Cani PD, Mayer EA. 2016. Gut microbiome and liver diseases. Gut 65:2035–2044. doi:10.1136/gutjnl-2016-312729. 27802157

[16] Roumans KHM, Basset Sagarminaga J, Peters HPF, Schrauwen P, Schrauwen-Hinderling VB. 2021. Liver fat storage pathways: methodologies and dietary effects. Curr Opin Lipidol 32:9–15. doi:10.1097/MOL.0000000000000720. 33234776 PMC7810416

[17] Wang W, Xu AL, Li ZC, Li Y, Xu SF, Sang HC, Zhi F. 2020. Combination of probiotics and *Salvia miltiorrhiza* polysaccharide alleviates hepatic steatosis via gut microbiota modulation and insulin resistance improvement in high fat-induced NAFLD mice. Diabetes Metab J 44:336–348. doi:10.4093/dmj.2019.0042. 31950772 PMC7188963

[18] Sun S, Araki Y, Hanzawa F, Umeki M, Kojima T, Nishimura N, Ikeda S, Mochizuki S, Oda H. 2021. High sucrose diet-induced dysbiosis of gut microbiota promotes fatty liver and hyperlipidemia in rats. J Nutr Biochem 93:108621. doi:10.1016/j.jnutbio.2021.108621. 33705945

[19] Ke X, Walker A, Haange SB, Lagkouvardos I, Liu Y, Schmitt-Kopplin P, von Bergen M, Jehmlich N, He X, Clavel T, Cheung PCK. 2019. Synbiotic-driven improvement of metabolic disturbances is associated with changes in the gut microbiome in diet-induced obese mice. Mol Metab 22:96–109. doi:10.1016/j.molmet.2019.01.012. 30792016 PMC6437638

[20] Zhang Y, Du R, Wang L, Zhang H. 2010. The antioxidative effects of probiotic *Lactobacillus casei* Zhang on the hyperlipidemic rats. Eur Food Res Technol 231:151–158.

[21] Liang X, Zhang Z, Zhou X, Lu Y, Li R, Yu Z, Tong L, Gong P, Yi H, Liu T, Zhang L. 2020. Probiotics improved hyperlipidemia in mice induced by a high cholesterol diet via downregulating FXR. Food Funct 11:9903–9911. doi:10.1039/D0FO02255A. 33094788

[22] Hou Q, Zhao F, Liu W, Lv R, Khine WWT, Han J, Sun Z, Lee YK, Zhang H. 2020. Probiotic-directed modulation of gut microbiota is basal microbiome dependent. Gut Microbes 12:1736974. doi:10.1080/19490976.2020.1736974. 32200683 PMC7524168

[23] Holmes E, Li JV, Athanasiou T, Ashrafian H, Nicholson JK. 2011. Understanding the role of gut microbiome–host metabolic signal disruption in health and disease. Trends Microbiol 19:349–359. doi:10.1016/j.tim.2011.05.006. 21684749

[24] Yan S, Chen J, Zhu L, Guo T, Qin D, Hu Z, Han S, Zhou Y, Akan OD, Wang J, Luo F, Lin Q. 2022. Oryzanol attenuates high fat and cholesterol diet-induced hyperlipidemia by regulating the gut microbiome and amino acid metabolism. J Agric Food Chem 70:6429–6443. doi:10.1021/acs.jafc.2c00885. 35587527

[25] Liu H, Pan LL, Lv S, Yang Q, Zhang H, Chen W, Lv Z, Sun J. 2019. Alterations of gut microbiota and blood lipidome in gestational diabetes mellitus with hyperlipidemia. Front Physiol 10:1015. doi:10.3389/fphys.2019.01015. 31447702 PMC6691352

[26] Wastyk HC, Fragiadakis GK, Perelman D, Dahan D, Merrill BD, Yu FB, Topf M, Gonzalez CG, Van Treuren W, Han S, Robinson JL, Elias JE, Sonnenburg ED, Gardner CD, Sonnenburg JL. 2021. Gut-microbiota-targeted diets modulate human immune status. Cell 184:4137–4153.e14. doi:10.1016/j.cell.2021.06.019. 34256014 PMC9020749

[27] Lee J-B, Ahn H, Kwaon S-M, Kim M-J. 2022. Research on hyperlipidemia improvement and diet fresh convenience HMR product development. The Korean J Food and Nutrition 35:159–166.

[28] McFarland LV, Goh S. 2019. Are probiotics and prebiotics effective in the prevention of travellers' diarrhea: a systematic review and meta-analysis. Travel Med Infect Dis 27:11–19. doi:10.1016/j.tmaid.2018.09.007. 30278238

[29] Lu D, Yan J, Liu F, Ding P, Chen B, Lu Y, Sun Z. 2019. Probiotics in preventing and treating chemotherapy-induced diarrhea: a meta-analysis. Asia Pac J Clin Nutr 28:701–710. 31826366 10.6133/apjcn.201912_28(4).0005

[30] Lai HH, Chiu CH, Kong MS, Chang CJ, Chen CC. 2019. Probiotic *Lactobacillus casei*: effective for managing childhood diarrhea by altering gut microbiota and attenuating fecal inflammatory markers. Nutrients 11:1150. doi:10.3390/nu11051150. 31126062 PMC6566348

[31] Liu A, Ma T, Xu N, Jin H, Zhao F, Kwok LY, Zhang H, Zhang S, Sun Z. 2021. Adjunctive probiotics alleviates asthmatic symptoms via modulating the gut microbiome and serum metabolome. Microbiol Spectr 9:e0085921. doi:10.1128/Spectrum.00859-21. 34612663 PMC8510161

[32] Chen M, Guo WL, Li QY, Xu JX, Cao YJ, Liu B, Yu XD, Rao PF, Ni L, Lv XC. 2020. The protective mechanism of *Lactobacillus plantarum* FZU3013 against non-alcoholic fatty liver associated with hyperlipidemia in mice fed a high-fat diet. Food Funct 11:3316–3331. doi:10.1039/C9FO03003D. 32226996

[33] Bene J, Hadzsiev K, Melegh B. 2018. Role of carnitine and its derivatives in the development and management of type 2 diabetes. Nutr Diabetes 8:8. doi:10.1038/s41387-018-0017-1. 29549241 PMC5856836

[34] Seccombe DW, James L, Hahn P, Jones E. 1987. l-carnitine treatment in the hyperlipidemic rabbit. Metabolism 36:1192–1196. doi:10.1016/0026-0495(87)90247-2. 3683188

[35] Gessner DK, Schwarz A, Meyer S, Wen G, Most E, Zorn H, Ringseis R, Eder K. 2019. Insect meal as alternative protein source exerts pronounced lipid-lowering effects in hyperlipidemic obese Zucker rats. J Nutr 149:566–577. doi:10.1093/jn/nxy256. 30726942

[36] Burlina AP, Sershen H, Debler EA, Lajtha A. 1989. Uptake of acetyl-l-carnitine in the brain. Neurochem Res 14:489–493. doi:10.1007/BF00964865. 2747840

[37] Virmani MA, Conti R, Spadoni A, Rossi S, Arrigoni-Martelli E. 1994. l-carnitine uptake into primary rat cortical cultures: interaction with GABA. Brain Res Mol Brain Res 25:105–112. doi:10.1016/0169-328X(94)90284-4. 7984034

[38] Yang C, Peng C, Jin H, You L, Wang J, Xu H, Sun Z. 2021. Comparison of the composition and function of the gut microbiome in herdsmen from two pasture regions, Hongyuan and Xilingol. Food Sci Nutr 9:3258–3268. doi:10.1002/fsn3.2290. 34136190 PMC8194741

[39] Truong DT, Franzosa EA, Tickle TL, Scholz M, Weingart G, Pasolli E, Tett A, Huttenhower C, Segata N. 2015. MetaPhlAn2 for enhanced metagenomic taxonomic profiling. Nat Methods 12:902–903. doi:10.1038/nmeth.3589. 26418763

[40] Li D, Liu CM, Luo R, Sadakane K, Lam TW. 2015. MEGAHIT: an ultra-fast single-node solution for large and complex metagenomics assembly via succinct de Bruijn graph. Bioinformatics 31:1674–1676. doi:10.1093/bioinformatics/btv033. 25609793

[41] Kang DD, Li F, Kirton E, Thomas A, Egan R, An H, Wang Z. 2019. MetaBAT 2: an adaptive binning algorithm for robust and efficient genome reconstruction from metagenome assemblies. PeerJ 7:e7359. doi:10.7717/peerj.7359. 31388474 PMC6662567

[42] Nissen JN, Johansen J, Allesøe RL, Sønderby CK, Armenteros JJA, Grønbech CH, Jensen LJ, Nielsen HB, Petersen TN, Winther O, Rasmussen S. 2021. Improved metagenome binning and assembly using deep variational autoencoders. Nat Biotechnol 39:555–560. doi:10.1038/s41587-020-00777-4. 33398153

[43] Wu YW, Simmons BA, Singer SW. 2016. MaxBin 2.0: an automated binning algorithm to recover genomes from multiple metagenomic datasets. Bioinformatics 32:605–607. doi:10.1093/bioinformatics/btv638. 26515820

[44] Sieber CMK, Probst AJ, Sharrar A, Thomas BC, Hess M, Tringe SG, Banfield JF. 2018. Recovery of genomes from metagenomes via a dereplication, aggregation and scoring strategy. Nat Microbiol 3:836–843. doi:10.1038/s41564-018-0171-1. 29807988 PMC6786971

[45] Parks DH, Imelfort M, Skennerton CT, Hugenholtz P, Tyson GW. 2015. CheckM: assessing the quality of microbial genomes recovered from isolates, single cells, and metagenomes. Genome Res 25:1043–1055. doi:10.1101/gr.186072.114. 25977477 PMC4484387

[46] Olm MR, Brown CT, Brooks B, Banfield JF. 2017. dRep: a tool for fast and accurate genomic comparisons that enables improved genome recovery from metagenomes through de-replication. ISME J 11:2864–2868. doi:10.1038/ismej.2017.126. 28742071 PMC5702732

[47] Chen W, Gong L, Guo Z, Wang W, Zhang H, Liu X, Yu S, Xiong L, Luo J. 2013. A novel integrated method for large-scale detection, identification, and quantification of widely targeted metabolites: application in the study of rice metabolomics. Mol Plant 6:1769–1780. doi:10.1093/mp/sst080. 23702596

